# Home Artificial Nutrition in Polish Children: An Analysis of 9-Year National Healthcare Provider Data

**DOI:** 10.3390/nu13031007

**Published:** 2021-03-21

**Authors:** Karolina Wyszomirska, Adam Wyszomirski, Michał Brzeziński, Anna Borkowska, Maciej Zagierski, Jarosław Kierkuś, Janusz Książyk, Hanna Romanowska, Magdalena Świder, Ewa Toporowska-Kowalska, Agnieszka Szlagatys-Sidorkiewicz

**Affiliations:** 1 Department of Pediatrics, Gastroenterology, Allergology and Nutrition, Faculty of Medicine, Medical University of Gdańsk, 80-803 Gdansk, Poland; brzezinski@gumed.edu.pl (M.B.); andzia@gumed.edu.pl (A.B.); zyga2809@gumed.edu.pl (M.Z.); agnieszka.szlagatys-sidorkiewicz@gumed.edu.pl (A.S.-S.); 2 Department of Adult Neurology, Faculty of Medicine, Medical University of Gdańsk, 80-211 Gdansk, Poland; adam.wyszomirski@gumed.edu.pl; 3 Department of Gastroenterology, Hepatology and Feeding Disorders, The Children’s Memorial Health, Institute, 04-730 Warsaw, Poland; j.kierkus@med-net.pl; 4 Department of Pediatrics, Nutrition and Metabolic Disorders, The Children’s Memorial Health Institute, 04-730 Warsaw, Poland; j.ksiazyk@ipczd.pl; 5 Department of Pediatrics, Endocrinology, Diabetology, Metabolic Diseases and Cardiology, Pomeranian Medical University, 71-252 Szczecin, Poland; hanna.romanowska@wp.pl; 6 Department of Anesthesiology and Critical Care Medicine, Clinical Provincial Hospital No. 2 in Rzeszow, 35-301 Rzeszow, Poland; madlenka.s@gazeta.pl; 7 Department of Pediatric Allergology, Gastroenterology and Nutrition, Medical University of Lodz, 91-738 Lodz, Poland; etka@op.pl

**Keywords:** home artificial nutrition, home enteral nutrition, home parenteral nutrition

## Abstract

Background: Home artificial nutrition (HAN) is a developing method of treatment that reduces the need for hospitalizations. The epidemiology of pediatric HAN in Poland has not yet been covered in detail. This study is a longitudinal nationwide analysis of incidence, prevalence, and patients’ profile for HAN in Polish children. Methods: Assessment of National Health Fund (NFZ) data covering all pediatric patients treated with HAN in Poland between 2010 and 2018. Results: HAN was received by 4426 children, 65 patients were on home enteral nutrition (HEN) or home parenteral nutrition (HPN) at different times (HEN *n* = 3865, HPN *n* = 626). HAN was most frequently started before the child was 3 years old and long-term HAN programs (5–9 years) were reported. The most common principal diagnosis in HEN was food-related symptoms and signs. In HPN, it was postoperative gastrointestinal disorders. A regionally differentiated prevalence of HAN patients and centers was demonstrated. Mortality among patients was 24.9% for HEN, and 9.6% for HPN, and the main in-hospital cause of death was cardiac arrest. Conclusions: HAN’s use is increasing and evolving in Poland. Uneven distribution of patients and centers results in difficult access to the nutritional procedure which, together with the increasing number of patients, highlights the need for data analysis and development of nutrition centers.

## 1. Introduction

Home artificial nutrition (HAN) includes procedures that provide specialist nutritional treatment in the home setting. HAN includes home enteral nutrition (HEN) and home parenteral nutrition (HPN). Predominant indications for HEN in pediatric patients include oral food supply disorders (predominantly the sucking and swallowing functions), disorders of digestion, absorption, gastrointestinal motility, and increased nutrient requirements or loss in chronic diseases [1,2,3]. The most common indications for HPN in children are gastrointestinal diseases associated with partial or total intestinal resection (short bowel syndrome), congenital bowel defects or motility disorders, severe congenital heart defects, and states of increased energy and nutrient requirements [4,5,6]. Nutritional treatment of the child at home reduces expenditure in the medical services sector by reducing the cost of long-term hospitalizations [5,7,8,9,10,11,12,13]. Globally, there has been a steady increase in the number of patients receiving home-delivered nutrition due to the increased availability and improvement of procedures [10,14], the inclusion of HAN in reimbursement [10,15,16], and the formation of multispecialty care teams [10].

Nutritional treatment registries allow monitoring and analysis of indications, complications, patient profile, and types of therapeutic methods used. Worldwide, HAN registries are maintained by Australia, Germany, Italy, Japan, Spain, Sweden, the United States, and the United Kingdom. Registries are mainly established at national gastroenterology and clinical nutrition societies [17]. In Europe, Spain has taken the lead in maintaining registries, with the registry of the group of The Home and Ambulatory Artificial Nutrition of Spanish Society of Enteral and Parenteral Nutrition (NADYA-SENPE) reporting data on adult and pediatric patients <14 years of age since 1992, and the Spanish national registry for pediatric home enteral nutrition (NEPAD), which has collected data on pediatric patients since 2003 [10,12]. The UK has maintained the information on trends in HAN from the British Artificial Nutrition Survey (BANS) established by the British Association for Parenteral and Enteral Nutrition (BAPEN) since 1996 [18]. Data on the prevalence of pediatric HAN in Italy come from a 2016 survey, which gave rise to a national HAN registry in 2017 [7]. Current registries are based on data that were voluntarily uploaded into systems. The information covers some, but not all, HAN patients and is entered by those directly involved in the care of nutritional patients. To date, no studies have been published analyzing and summarizing data on all patients receiving refinanced, home-based nutritional support in any country during a long-term follow-up. In Poland, home feeding procedures are funded by a single payer: the National Health Fund. The HPN procedure has been reimbursed since 1997, and the HEN procedure has been reimbursed since 2007. Poland does not have a registry of data on enteral nutrition patients. However, analysis of data from the single payer financing HAN allows for the assessment of all patients undergoing the procedure, at least in the scope of information collected in the National Health Fund system. The only analysis published to date focused exclusively on enteral nutrition (HEN) in Poland and dates from the initial period of reimbursement of the procedure by the National Health Fund (NFZ), which was in 2010 [11]. The aim of this study is to assess the frequency, prevalence, and profile of pediatric patients undergoing HAN in Poland.

## 2. Materials and Methods

This study is a retrospective analysis of pediatric patients who underwent home enteral and parenteral nutritional procedures in Poland between 2010 and 2018. All public-financed medical centers that provide care for patients requiring home enteral nutritional treatment report their services to regional branches of the National Health Fund. The reimbursement rules for HEN and HPN procedures remain the same for all centers. The data presented were obtained from the National Health Fund. These are anonymized data on all pediatric patients who underwent the HEN and HPN procedure at regional centers between 1 January 2010 and 31 December 2018. A computerized database was assessed. The analysis included determining the number of patients aged 0–18 years undergoing HEN and HPN procedures between 2010 and 2018, highlighting the changing annual trends. The age range and principal diagnosis of patients at the time they received their first nutritional service was examined. Additionally, the analysis included the most common comorbid diagnoses, the length of time the patients have been undergoing HEN and HPN (“duration”), and the geographical distribution of patients. The number and types of centers providing nutritional treatment were described. Mortality among patients with HAN, the number of deaths, and their in-hospital causes during the study period were analyzed. Categorical data were presented as counts and percentages. The analyzed data quality was verified in accordance with the criteria set out in Ordinances No. 23/2009/DSOZ, 55/2014/DSOZ, 60/2015/DK, 45/2016/DK, 128/2017/DK of the President of the National Health Fund concerning control activities carried out by the National Health Fund [19].

Statistical calculations were performed and figures and graphs were generated in the statistical software R version 3.6.3.

## 3. Results

In the period from 1 January 2010 to 31 December 2018, 4426 patients aged 0–18 years underwent the procedure of home enteral and parenteral nutrition in Poland.

In this group, 65 (1.5%) children received both HEN and HPN treatment at different times during the analyzed period. The total number of patients reported to the National Health Fund by regional centers between 2010 and 2018, for both procedures, amounted to 4491. HEN represented 86.1% and HPN represented 13.9%, respectively.

**Figure 1:**
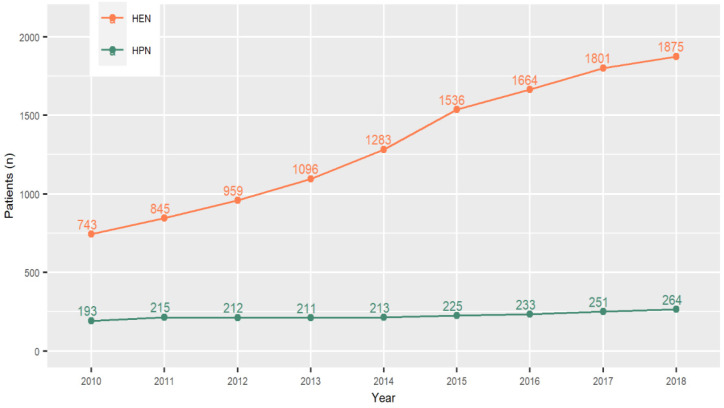
The number of patients undergoing home enteral nutrition (HEN) and home parenteral nutrition (HPN) between 2010 and 2018.

During the 9-year follow-up period (comparing the number of patients treated with both nutritional procedures in 2010 and 2018), there was a 2.5-fold increase in the number of patients treated with HEN and a 1.4-fold increase in the number of patients on the HPN procedure. Figure 1 shows the number of HEN and HPN patients in each year.

Patients undergoing HAN over a multiyear period are counted in all relevant annual summaries.

Table 1 shows the age distribution of patients, taking into account the age of the patient (by year of birth) at the start of the home enteral and parenteral nutritional procedure.

The principal diagnoses of HEN and HPN are presented in Table 2 and Table 3.

Comorbid diagnoses were reported for 1742 patients with HEN and for 273 patients with HPN. For HEN, the most common comorbid diagnoses were epilepsy (11.3%), infantile cerebral palsy (10.9%), dysphagia (10.4%), and care of patients with artificial orifices (9.6%). For HPN, postoperative gastrointestinal disorders (35.5%), congenital intestinal malformations (11.7%), conditions related to the presence of an artificial orifice (7.3%), and intestinal malabsorption (5.1%) were the most common comorbid diagnoses. The duration of treatment was analyzed for patients whose nutritional procedures began in 2010. The observation covered a 9-year period from 1 January 2010 to 31 December 2018 (Table 4).

**Figure 2:**
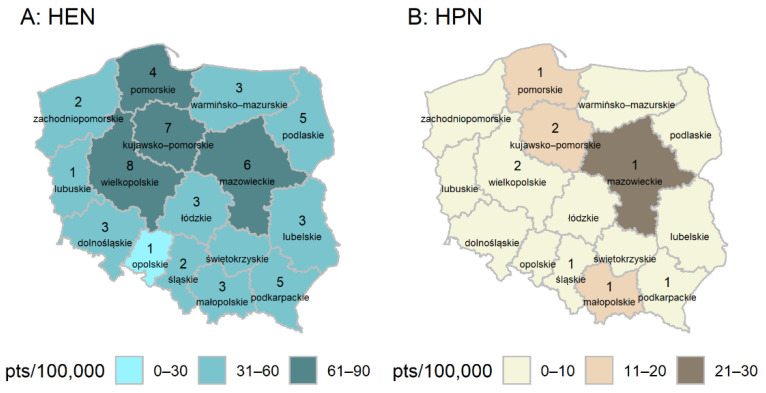
The number of patients treated with (**A**) HEN and (**B**) HPN between 2010 and 2018 per 100,000 underage population, and number of centers by voivodeship.

Figure 2 shows the number of pediatric HEN and HPN patients per 100,000 patients under 18 in a voivodeship (a region in Poland, Nomenclature of Territorial Units for Statistics (NUTS) code level 2, equivalent to “province”) and the number of nutrition centers. The total number of patients within voivodeships is higher than the total reported number of patients on HEN and HPN procedures, which is due to migration of patients between voivodeships.

A total of 57 centers provided HAN (48 HEN, 8 HEN and HPN, 1 HPN). These centers included hospitals with in-hospital nutrition clinics (*n* = 22, 38.6%), hospices (*n* = 7, 12.3%), and the category “other”, which included: non-public health care facilities, private medical centers, out-of-hospital nutrition clinics, and specialist health care facilities (*n* = 28, 49.1%). The Children’s Memorial Health Institute (Centrum Zdrowia Dziecka (CZD)) in Warsaw provided care for the largest group of HAN patients in the study period (reporting HEN *n* = 867 and HPN = 386).

The prevalence of HEN (number of patients/million population under 18 years of age) in 2010 was 104.1 patients/million, increasing to 270.3 patients/million in 2018.

The prevalence of HPN (number of patients/million population under 18 years of age) in 2010 was 27 patients/million, increasing to 38.1 patients/million in 2018.

In the total group of 4426 patients, 1021 children died. The reported mortality rate was 24.9% for patients undergoing HEN and 9.6% for patients undergoing HPN. Data on in-hospital causes of death were obtained (Table 5), concerning 458 HEN patients and 41 HPN patients. The main in-hospital cause of death for children with HAN was cardiac arrest.

## 4. Discussion

The principles of uniform HAN reimbursement for nutrition providers and reporting of services to a single payer allowed us to obtain data on all pediatric patients treated with HEN and HPN between 2010 and 2018. Thus, this study represents the first analysis that includes all pediatric patients treated with nutrition (HEN and HPN) at home between 2010 and 2018 in Poland. According to our knowledge, this is the first study of its kind in Europe. The existing registries provide information on an incomplete number of patients on home artificial nutrition, due to voluntary reporting of data. A gradual increase in the number of centers and specialists collaborating with European registries has been observed [20,21]. Nevertheless, in the periods analyzed, the data do not cover all patients. A variety of demographic and epidemiological data are gathered in the registries, some of which are common to the data of this analysis (number of patients, age at onset of the procedure, principal diagnosis, number of centers, prevalence, and length of time undergoing the HAN procedure) [7,10,18,22]. In European registries, data directly related to the procedure (type of diet used, infusion regimen, gastrointestinal access route) are reported in detail [7,10,15,18,22,23]. Our data are limited to other variables due to the nature of the information reported to the National Health Fund. However, to the extent available, our data cover the entire treated pediatric population, which allows us to fully estimate the prevalence of HEN and HPN in children in Poland.

This study showed a gradually increasing number of patients on HAN, with a total of 4,426 patients recorded, among whom HEN was the main type of home nutrition. An increasing number of patients on HAN has also been observed in Italy, Spain, France, the United Kingdom, and the United States [7,10,15,18,22,24,25,26].

The last analysis of HEN epidemiology in Poland in 2010 showed 525 HEN patients [11], who represented 70.6% of the actual number of HEN patients in 2010 (*n* = 743, 100%). This study involved a much larger group of children, which indicates a dynamic increase in the number of HEN patients.

A smaller number of HEN patients, 952 between 2003 and 2010, was reported in Spain, but a larger than 25-fold increase in the number of patients [10] was observed. In 2018, 23 HPN patients were reported in Spain, while in the same year, 264 patients were reported to receive HPN in Poland [20]. The Italian analysis reported 2,277 children with HEN and 179 with HPN [7]. In the same year (2016) in Poland, there were 1,664 HEN patients and 233 HPN patients, respectively. In the USA in 2013, the estimated number of patients with HEN was 189,036, and the estimated number with HPN was 4129 [24].

The prevalence of children on HEN (number of patients/million population under 18 years of age) in Poland in 2010 was 104.1 patients/million, increasing to 270.3 patients/million in 2018. The prevalence in the Italian pediatric population (the Italian pediatric population includes individuals up to the age of 19 years) in 2016 was 204.7 patients/million for HEN and 15.8 patients/million for HPN [7]. In the same year, the prevalence of HAN in the Polish pediatric population was 241.3 patients/million for HEN and 33.8 patients/million for HPN. On the other hand, the Spanish authors emphasized that the voluntary reporting to their national registries did not allow for the full assessment of HEN prevalence [10].

In Poland, the HAN procedure was most frequently initiated in the first 3 years of the child’s life. The observation is consistent with European data; the median age in Italy was 2.3 years for HEN patients and 0.8 years for HPN patients [7], in Spain it was 1.6 years for HEN [10] respectively, while in the UK, children under 2 years of age accounted for approximately 69% of HEN patients [18]. Early French observations recorded patients beginning HEN at the mean age of 5.4 years old and a decreasing trend. This likely resulted from the earlier recognition of the need for nutritional support in chronic disease and the development of gastrostomy care for infants and younger children [15]. In enteral nutrition, adolescents represented the second largest group of patients, which seems to result from the increasing survival rate of children with chronic diseases of the nervous system [27], who represent a significant proportion of HEN beneficiaries [7,10,11,15,18,22].

Analysis of the principal diagnoses in HEN identified symptoms and signs concerning food and fluid intake as the main diagnoses in that subgroup, and in patients qualified for the HPN procedure, it was postoperative gastrointestinal disorders. Comparison of principal diagnoses in the Polish pediatric population with data from other countries was made difficult by the interchangeable reporting of diagnoses and indications as “principal diagnoses” to the National Health Fund. In Spanish data, indications were separated from principal diagnoses when analyzing HEN applications. Reduced oral food intake, which is the leading principal diagnosis in this analysis (symptoms and signs concerning food and fluid intake), was the main indication in the Spanish population (64%) [10]. If this diagnosis was taken as an indication for HEN initiation, then the diseases of the central nervous system, leading in European registries (Italy approx. 65%, Spain 30.5%, France 35%, UK 30%) [7,10,15,18], would be the most common principal diagnosis of this analysis.

HEN patients in our study showed a lower proportion of gastrointestinal diseases compared to other countries (Italy approx. 18%, Spain 18.3%, France 35%), but a higher proportion of diseases related to nutritional and metabolic disorders (Italy <5%, Spain 13.1%). In accordance with Italy, HEN is used in a small proportion of oncology patients (Poland 0.6%, Italy approx. 1%), which were more frequently observed in Spain (15.3%), France (11%), and the UK (6.2–11%) [7,10,15,18].

A comparison of principal diagnoses determined for the HEN population under analysis with diagnoses from Polish studies from 2010 and 2016 allowed us to assess the changing trends of HEN in Poland [11,28]. In 2010, higher numbers of children with diseases of the central nervous system (CNS) (64.2% vs. 21.9%), and similar numbers of patients with diseases of the digestive system (6% vs. 7.7%) and neoplasms (1% vs. 0.6%), were reported [11].

In the study of a 6-year experience in HEN from a leading nutrition center in Poland (CZD), CNS diseases accounted for 48% of diagnoses [28]. 

In this analysis respiratory (0.8%) and cardiovascular (0.2%) diseases are less frequently recorded than in European registries [7,10]. It would appear that predominantly malnutrition resulting from these diseases is reported in Poland.

The described distinctions in the principal diagnoses in HEN patients highlight the need for standardized and clear registries that not only enable comparative analysis, but above all can contribute to the appropriate qualification for nutritional treatment of patients with chronic diseases. In HPN, postoperative gastrointestinal disorders were the main principal diagnosis in practice associated with the development of short bowel syndrome [29,30], which was the most common principal diagnosis in HPN patients in Italy (approx. 80%), Spain (60.9%), the United Kingdom (63%), and France (40.8%) [7,20,25,26].

Patients were shown to undergo HAN procedures for long periods of time (for a period of 5–9 years). Long-term programs were also reported in Italy, where the average duration of HEN treatment was 6 years, and for HPN, the average duration was 3 years [7]. With a relatively large number of HAN patients aged 14–19 years old, this observation highlights the need for continuity and organization of care for nutritional patients following their transition to the adult medical services sector. Poorly planned transitions can cause difficulties in accessing specialist adult medical services [30]. Successful transition to adult medical facilities requires careful planning and a multidisciplinary approach to the patient [31,32].

The assessment of HAN prevalence in different regions of the country was limited by migration of patients between voivodeships. Patients who moved were reported several times within different regions of the country (the total number reported was 4766). An approximate analysis showed an uneven distribution of HAN patients and centers in Poland. Recent data on HEN in the pediatric population from 2010 also shows an uneven distribution of patients across the country [11], including no patients in the Lubuskie and Opolskie Voivodeships. The current analysis shows the presence of HEN and HPN patients in all voivodeships. From 2010, the number of HEN centers increased (from 14 to 57) [11], whereas their distribution across the country, in a similar vein to the prevalence of patients, remained uneven. Centers providing enteral nutrition services to patients were located in all voivodeships apart from the Świętokrzyskie Voivodeship, with the highest number of centers in the Wielkopolskie Voivodeship (*n* = 8).

The distribution of patients with HEN was in the range of 0–30 patients to 61–90 patients per 100,000 patients under 18, and no direct relationship was observed between the number of patients per voivodeship and the number of centers. Similar regional differences in the distribution of HEN in children were previously reported in Spain and Italy [7,33].

The continued uneven prevalence of HAN patients [11] and centers results in difficulties in access to nutritional treatment in some regions of the country. This observation, with the continuously increasing number of HAN patients, highlights the importance of the analysis of nutritional patient data, education, and development in centers. These actions aim to not only improve the treatment methods used, but, most importantly, to increase patient access to multispecialty therapy.

## 5. Conclusions

HAN is actively used and is continuously developing as a treatment approach in Poland, especially as home enteral nutrition. The observations concerning the increasing number of HAN programs, the young age of patients on HAN initiation, the long-term programs are consistent with data from other European countries and highlight the need for specialist management of home enteral and parenteral nutrition. In addition to the dynamic development of pediatric HAN services, uneven distribution of patients and HAN centers across our country has been demonstrated, and potential regional shortages have been documented. These result in difficulties in accessing nutritional procedures for some patients in Poland. This finding, together with the continuously increasing number of patients with HAN, highlights the need for data analysis, education, and further development of nutrition centers.

The increasing number of nutrition centers in Poland results in an increase in the number of patients, especially those treated with HEN; however, the issue of unequal access requires effective resolution. The creation of a center network registry could not only improve care, but also increase patient access to the nutritional procedure in Poland. 

## Figures and Tables

**Table 1:** Patient age by year of birth at the start of HEN and HPN procedures.

Age Years	Enteral Nutrition (*n* = 3865)	Parenteral Nutrition (*n* = 626)
0–2	1300 (33.6%)	424 (67.7%)
3–5	570 (14.7%)	79 (12.6%)
6–9	571 (14.8%)	48 (7.7%)
10–13	626 (16.2%)	29 (4.6%)
14–19	798 (20.6%)	46 (7.3%)

**Table 2:** Principal diagnoses in HEN patients by ICD-10 groups.

Diagnosis Related Group	*n* (%) ^1^
**Symptoms, pathological features, and abnormal findings, not elsewhere classified**	**914 (23.6%)**
Symptoms and signs concerning food and fluid intake	604 (15.6%)
Dysphagia	147 (3.8%)
Lack of expected normal physical development	129 (3.3%)
Unknown and unspecified causes of morbidity	24 (0.6%)
Other	10 (0.3%)
**Diseases of the nervous system**	**848 (21.9%)**
Infantile cerebral palsy	457 (11.8%)
Other disorders of brain	130 (3.4%)
Spinal muscular atrophy and related syndromes	87 (2.3%)
Paraplegia (paraparesis) and quadriplegia (quadriparesis)	43 (1.1%)
Epilepsy	39 (1.0%)
Primary disorders of muscles	30 (0.8%)
Other	62 (1.7%)
**Endocrine, nutritional, and metabolic diseases**	**845 (21.9%)**
Cachexia	348 (9.0%)
Protein-energy malnutrition of moderate and mild degree	181 (4.7%)
Cystic fibrosis	81 (2.1%)
Unspecified protein-calorie malnutrition	77 (2.0%)
Disorders of sphingolipid metabolism and other lipid storage disorders	52 (1.3%)
Unspecified severe protein-calorie malnutrition	39 (1.0%)
Other metabolic disorders	26 (0.7%)
Nutritional and metabolic disorders in diseases not elsewhere classified	27 (0.7%)
Other	14 (0.4%)
**Factors influencing health status and contact with health services**	**332 (8.6%)**
Encounter for attention to artificial openings	290 (7.5%)
Artificial opening status	42 (1.1%)
**Diseases of the digestive system**	**302 (7.7%)**
Crohn’s disease /regional enteritis	187 (4.8%)
Gastro-esophageal reflux disease	52 (1.3%)
Other	63 (1.6%)
**Congenital malformations, deformations, and chromosomal abnormalities**	**177 (4.6%)**
Other specified congenital malformation syndromes affecting multiple systems	38 (1.0%)
Congenital malformations of esophagus	26 (0.7%)
Other congenital malformations of brain	23 (0.6%)
Edwards’ syndrome and Patau’s syndrome	23 (0.6%)
Other congenital malformations, not elsewhere classified	21 (0.5%)
Other	46 (1.3%)
**Diseases of the respiratory system**	**32 (0.8%)**
Respiratory failure, not elsewhere classified	32 (0.8%)
**Neoplasms**	**25 (0.6%)**
Malignant neoplasm of brain	25 (0.6%)
Other *	**390 (10.1%)**

* The other group includes other diagnoses that involved fewer than 20 patients with HEN; ^1^ Percentages may not add up to 100% due to rounding.

**Table 3:** Principal diagnoses in HPN patients by ICD-10 groups.

Diagnosis Related Group	*n* (%) ^1^
**Diseases of the digestive system**	**494 (78.9%)**
Postprocedural disorders of digestive system, not elsewhere classified	464 (74.1%)
Other functional intestinal disorders	16 (2.6%)
Intestinal malabsorption	14 (2.2%)
**Endocrine, nutritional, and metabolic diseases**	**21 (3.3%)**
Protein-energy malnutrition of moderate and mild degree	12 (1.9%)
Unspecified protein-calorie malnutrition	9 (1.4%)
**External causes of morbidity and mortality, and factors influencing health status and contact with health services**	**18 (2.9%)**
Acquired absence of organs, not elsewhere classified	10 (1.6%)
Encounter for attention to artificial openings	8 (1.3%)
**Congenital malformations, deformations, and chromosomal abnormalities**	**12 (1.9%)**
Other congenital malformations of intestine	12 (1.9%)
Other *	**81 (12.9%)**

* Diagnoses that affected fewer than 8 HPN patients are included in the ‘other’ category; ^1^ Percentages may not add up to 100% due to rounding.

**Table 4:** Duration of treatment of patients starting the HEN and HPN procedures in 2010.

Duration of Treatment in Months	Enteral Nutrition (*n* = 743)	Parenteral Nutrition (*n* = 193)
0–5	215 (28.9%)	46 (23.8%)
6–11	116 (15.6%)	32 (16.6%)
12–23	72 (9.7%)	16 (8.3%
24–35	48 (6.5%)	14 (7.3%)
36–47	35 (4.7%)	5 (2.6%)
48–59	38 (5.1%)	12 (6.2%)
60–107	219 (29.5%)	68 (35.2%)

**Table 5:** Causes of in-hospital deaths for HEN and HPN patients within 365 days of service provision completion.

Diagnosis According to ICD 10	Enteral Nutrition (*n* = 961)	Parenteral Nutrition (*n* = 60)
No data available	503 (52.3%)	19 (31.7%)
Cardiac arrest	164 (17.1%)	16 (26.7%)
Respiratory failure, not elsewhere classified	74 (7.7%)	9 (15.0%)
Other specified symptoms and signs involving the circulatory and respiratory systems	61 (6.3%)	0 (0.0%)
Heart failure	57 (5.9%)	8 (13.3%)
Childhood cerebral palsy	15 (1.6%)	0 (0.0%)
Other disorders of brain	14 (1.5%)	0 (0.0%)
Shock, not elsewhere classified	13 (1.4%)	0 (0.0%)
Other sepsis	5 (0.5%)	0 (0.0%)
Bronchopneumonia, unspecified organism	8 (0.8%)	0 (0.0%)
Other *	47 (4.9%)	8 (13.3%)

* For diagnoses of fewer than 5 patients, the category ‘other’ was assigned.

## Data Availability

The data presented in this study are available on request from the corresponding author. The limited version of analyzed data are publicly available on the National Health Fund’s website [https://zdrowedane.nfz.gov.pl/course/view.php?id=27] (accessed on 11 September 2019).
